# Exosomal Non-coding RNAs-Mediated Crosstalk in the Tumor Microenvironment

**DOI:** 10.3389/fcell.2021.646864

**Published:** 2021-04-12

**Authors:** Qi Chen, Yuefeng Li, Yueqin Liu, Wenlin Xu, Xiaolan Zhu

**Affiliations:** ^1^Department of Oncology and Central Laboratory, Fourth Affiliated Hospital of Jiangsu University, Zhenjiang, China; ^2^Affiliated People Hospital of Jiangsu University, Zhenjiang, China; ^3^Reproduction Medicine Center, Fourth Affiliated Hospital of Jiangsu University, Zhenjiang, China; ^4^International Genome Center, Jiangsu University, Zhenjiang, China

**Keywords:** exosomes, non-coding RNAs, cancer, stromal cells, biological function

## Abstract

Exosomes are secreted by different types of cells in tumor microenvironment (TME) and participate in multiple biological processes of tumors. Non-coding RNAs (ncRNAs) enveloped in exosomes and released to the TME are shown to be involved in tumorigenesis and development, as well as act as important intracellular communication mediators. However, the understanding on the exact regulatory functions and substrates of exosomal RNA is still at an early stage. In this review, we provided an overview on recent studies on exosomes mediating the modulation of both tumor cells and immune cells, then summarized the exosomal ncRNAs [such as microRNAs (miRNAs), long non-coding RNAs (lncRNAs), and circular RNAs (circRNAs)] secreted by tumor cells and stromal cells that exhibited potential capabilities to regulate tumor cell growth, progression, metastasis, drug resistance, and immune response. Our review may hopefully inspire a deeper understanding on the ncRNAs’ function as useful biomarkers for the diagnosis, prognosis, and as novel targets therapy for cancer.

## Introduction

The tumor microenvironment (TME) is a complex ecosystem formed by cells of diverse types, including endothelial, fibroblastic, and immune cells, which participate in all stages of tumor initiation and progression (Meurette and Mehlen, 2018; Li and Nabet, 2019). A bidirectional communication exists between cancer cells and the TME, and such crosstalk involves the transportation of membrane-encapsulated particles containing functional molecules from cancer cells and/or stromal cells (Yuan et al., 2016; Xu et al., 2018). Indeed, by offering the inhibitory or stimulatory signals, the TME acts as an essential modulator in tumor development and progression and an effective source for the identification of potential therapeutic agents.

Exosomes have been investigated as crucial factors in mediating the communications between tumor and microenvironment-related cells, achieving intercellular transportation of substance and information, and modulating the immune system (Maia et al., 2018; Xie et al., 2019), and exosomes shedding from host cells have been found as another way of intercellular signal transmission (Quail and Joyce, 2013; Li and Nabet, 2019; Wortzel et al., 2019). The generated process of exosomes includes endocytosis, exocytosis, sorting, and transportation (Kalluri and LeBleu, 2020). Studies suggest that tumor cells or stromal cells release exosomes to coordinate cell behaviors in the intracellular communication by packing them with various growth factors, proteins, lipids, and nucleic acids, including microRNAs (miRNAs) (Fong et al., 2015), long non-coding RNAs (lncRNAs) (Zheng R. et al., 2018), and circular RNAs (circRNAs) (Wang et al., 2020), which are horizontally transferred from donor to recipient cells. In addition, exosomes play a similar role on antigen-presenting cells, and their surface contains a large number of molecules related to antigen presentation, which can induce immune response (Xu et al., 2018). Therefore, circulating exosomes have a potential in clinical application as precise carriers for the delivery of specific agents contributing to tumor progression, metastasis, immune regulation, angiogenesis, and tissue regeneration, as well as anticancer vaccines.

With the development of deep RNA sequencing (RNA-seq) technologies and novel bioinformatics methods, diverse ncRNAs, including miRNAs, transfer RNA-derived small RNAs (tsRNAs), PIWI-interacting RNAs (piRNAs), lncRNAs, pseudogenes, and circRNAs, have been detected and identified in exosomes. These ncRNAs are abundantly present in exosomes secreted by tumor cells (Felicetti et al., 2016; Li B. et al., 2018; Ma et al., 2020) and participate in various regulatory processes, such as chromatin modification, transcription activation, competitive splicing, and protein interaction, thereby contributing to cell proliferation and metastasis (Zhu L. et al., 2019). Interestingly, these functional ncRNAs enriched in the exosomes can be detected in the circulation, plasma, and urine. Studies suggest that plasma exosomal tsRNA (tRNA-ValTAC-3, tRNA-GlyTCC-5, tRNA-ValAAC-5, and tRNA-GluCTC-5) in hepatic carcinoma can serve as diagnostic biomarkers (Zhu L. et al., 2019); piRNAs [piR-004800 (Ma et al., 2020), piR-10506469, and piR-20548188 (Gu et al., 2020)] have exhibited crucial roles in tumorigenesis and progression of multiple myeloma. Furthermore, exosomes facilitate tumor initiation and development through modulating the TME. Circulating exosomes surrounding the TME regulate energy metabolism, provide stimulus or inhibitory signals, and promote the epithelial–mesenchymal transition (EMT), extracellular matrix (ECM) remodeling, and endothelial tubulogenesis, therefore resulting in tumor metastatic invasion, angiogenesis, and chemoresistance.

In this review, we described the important roles of cancer cell-derived exosomes in the modulation of TME, listed examples of different types of ncRNAs, and detailed their profound effects. Then, we focused on the crucial roles of exosomal ncRNAs derived from diverse stromal cells in the mediation of cancer phenotype and progression. We then also summarized the potential clinical applications of exosomes in the prognosis, diagnosis, and therapy in various types of cancer.

## The Tumor Microenvironment

**FIGURE 1:**
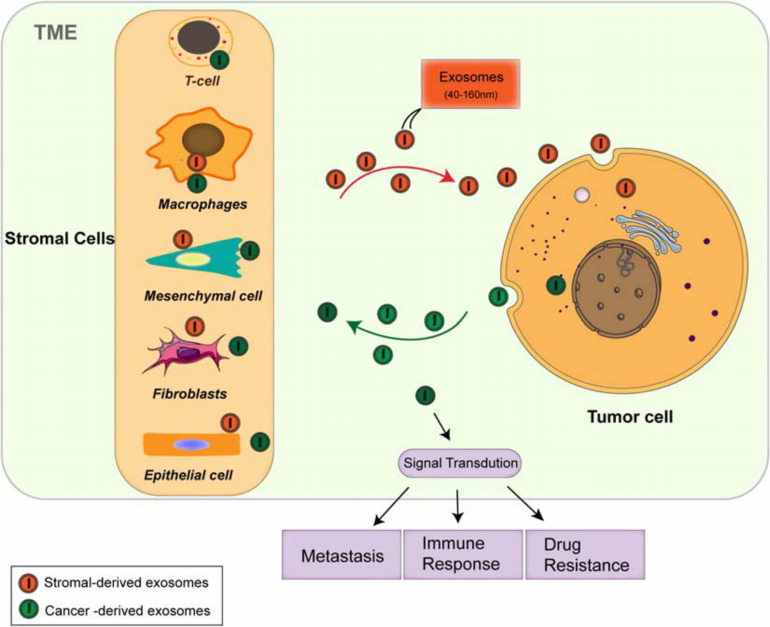
Both cancer cells and stromal cells release exosomes within the tumor microenvironment (TME). The stromal cells include cancer-associated fibroblasts (CAFs), endothelial cells, immune cells such as myeloid-derived suppressor cells (MDSCs), regulatory T cells (Tregs), and tumor-associated macrophages (TAMs). Both tumor cells and stromal cells release different types of the exosomes. The circulation of these exosome cargoes regulates tumor metastasis, immunoresponse, and chemoresistance through multiple signaling pathways.

The TME consists of various cellular and cell-free components, including types of stromal cells, such as cancer-associated fibroblasts (CAFs), endothelial cells, immune cells [myeloid-derived suppressor cells (MDSCs), regulatory T cells (Tregs), and tumor-associated macrophages (TAMs)] (Naito et al., 2017; Del Vecchio et al., 2018; Lambrechts et al., 2018), and mast cells; the cell-free components include blood and lymphatic vessels, extracellular matrix (ECM), and soluble proteins (Belli et al., 2018; Menon et al., 2019; Wu et al., 2019b). The TME is not a static entity but rather conditionally changing, especially in the development from the pre-tumor niche to invasive tumor (Belli et al., 2018). The dynamic and interactions among the component cells within the TME is essential for maintaining the characteristics of cancer cells and regulating cell transformation, proliferation, and metastasis (Wu et al., 2019b). Signal is transmitted between tumor cells and the TME by diverse cytokines (Bretz et al., 2013), growth factors (Hinshaw and Shevde, 2019), and hormones, through direct contact and/or different kinds of the extracellular vesicles (EVs) (Becker et al., 2016; Yekula et al., 2019; Jurj et al., 2020) [exosomes and microvesicles (Minciacchi et al., 2015)], resulting in the transformation through epithelial–mesenchymal transition (EMT) and angiogenesis, supporting the establishment of pre-metastatic niches and growth and metastasis of primary tumors. Exosomes have been explored as the key factors in mediating cell-to-cell communication between tumor cells and microenvironment, which takes part in various signaling pathways of modulation in the TME (Figure 1), providing novel methods for the treatment of antitumor immunity (Fanini and Fabbri, 2017), and have the potential abilities to modulate the TME and participate in tumor metastasis, immune response (Ruivo et al., 2017), and multidrug resistance (Skalska et al., 2019).

Increasing observations strongly indicate that the TME and stroma are the basic sources of tumor immunoescape and immunotherapies (Menon et al., 2019). However, dysregulated chemokines lead to the recruitment and accumulation of TAMs, immature dendritic cells (DC), and MDSC and the formation of immunosuppressive TME. Both tumor cells and TAMs secrete diverse molecules to suppress the activity of cytotoxic T lymphocytes (CTLs) and natural killer (NK)-mediated immunity. CAFs in the stroma are also pleiotropic in the secretion of chemokines, cytokines, and metabolites, regulating the anti-tumor immunity. Exosomes derived from tumor and stromal cells have been shown to involve in all stages of tumor development, regulating aggressive behavior of cancer cells through diverse signaling pathways (Luga et al., 2012; Li and Nabet, 2019; Figure 1). Due to their natural features, exosomes are extremely important for TME-dependent cancer prognosis and therapy. In order to fully understand the TME, close investigation on exosomes and their cargoes will be a promising way, which may further lead to the development of effective clinical applications.

## Exosome-Mediated Regulation of Tumor Biology and Immune Response in the Tumor Microenvironment

### Biogenesis and Composition of Exosomes

**FIGURE 2:**
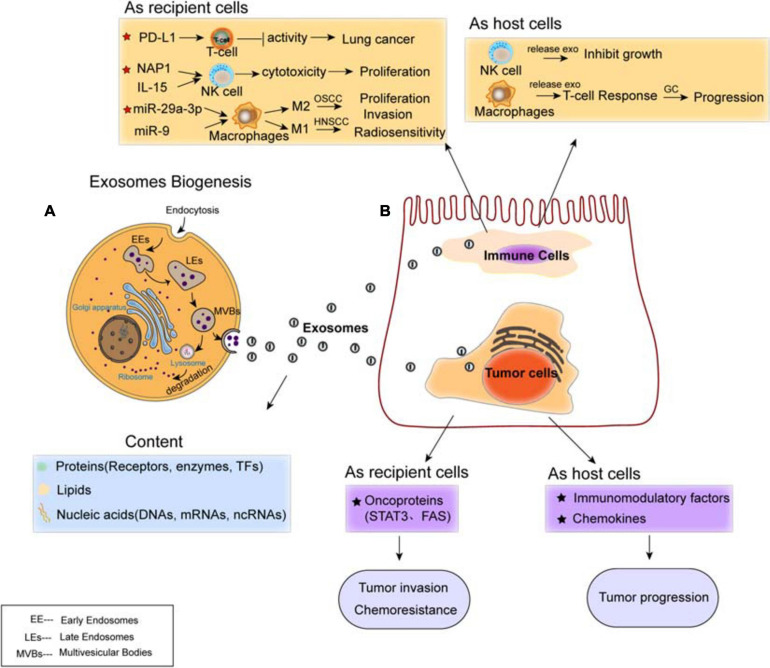
Exosomes mediate cell–cell communication between cancer cells and immune cells. **(A)** Biogenesis and contents of exosomes. The content of exosomes could be multiple proteins, lipids, and nucleic acids. **(B)** Regulation of exosomes on cancer cells and immune cells. Exosomal cargoes such as microRNAs (miRNAs) and proteins, which are derived from both immune cells (T-cell, NK-cell, and macrophages) and diverse types of cancer cells, can be transferred to recipient cells, thereby modulating the tumor proliferation, invasion, metastasis, and drug resistance. Both immune cells and cancer cells act as host cells and recipient cells.

Exosomes are classified as small membrane-bound extracellular vesicles (EVs), which are heterogeneous in size and components, present in cells and body fluids, released by fusion with the cell membrane, and deliver their cargoes from the donors to the recipients (Felicetti et al., 2016). The biogenesis of exosomes mainly includes three steps: first, the early endosomes (EEs) are generated by the fusion of endocytic vesicles and encapsulate the endocytic cargoes sharing certain components and membrane proteins; then, late endosomes (LE) are formed by inward budding of the multivesicular body (MVB) membrane. Invagination of LE membranes results in the formation of intraluminal vesicles (ILVs) within large MVBs. Third, MVBs enter the plasma membrane and release their component substance into the extracellular environment, which are considered as “exosomes” (Kalluri and LeBleu, 2020; Figure 2A). Alternatively, some of the MVBs may fuse with autophagosomes or lysosomes and then lead to ILVs’ degradation.

The formation of ILVs is mediated by endosomal-sorting complex required for transport (ESCRT), which is a machinery consists of four subcomplexes (ESCRT-0, I, II, and III) that function cooperatively to form MVBs, vesicle budding, and cargo sorting (He et al., 2018). The ESCRT complex is responsible for sorting of ubiquitinated proteins into ILV budding (Vietri et al., 2020). ESCRT-0 initially recognizes ubiquitinated proteins to specific domains of endosomal membrane, then ESCRT-I and ESCRT-II join the ESCRT-0 to make a strong recognition domain with high affinity to the ubiquitinated substrates where it will ultimately to form vesicle budding. Then, they combine with ESCRT-III and separate from the MVB membrane (Villarroya-Beltri et al., 2014; Vietri et al., 2020). Besides ESCRT, it has been shown that syndecan proteoglycans and their adaptor syntenin are also involving in endosomal membrane budding and abscission by interacting with marker protein ALIX (Baietti et al., 2012). Ceramide by neutral type II sphingomyelinase is delivered to ILVs in an ESCRT-independent manner, which emphasizes a crucial role of exosomal lipids in biogenesis. The tetraspanin (four-pass membrane protein) family proteins (such as CD63, CD81, and CD9) have been shown to regulate the sorting of vesicle contents involved in ESCRT-dependent and ESCRT-independent events (Kowal et al., 2014).

Cargo sorting is not a random event, which evolves to a certain extent with cell types and/or other biological alterations. Protein cargo packaging into exosomes is depended on ESCRT, tetraspanins, and lipids. Aside from exosomal proteins, it has been shown that sorting of RNA and miRNAs into exosomes can be regulated by the levels of miRNAs and endogenous target sequences (O’Brien et al., 2020). RNA-binding proteins that play a major role in intracellular RNA trafficking have been suggested to be involved in RNA sorting into exosomes (Zhang Y. et al., 2019). Thus, such communication predominantly involves the secretion of soluble factors by cancer cells and/or stromal cells within the TME (Xu et al., 2018). The process of encapsulating, exchanging, and releasing diverse contents realizes the exosome-mediated communication of multiple substances and signals, thereby inducing crucial signaling pathways in tumor cells and the TME for the establishment of the pre-metastatic niche and enhancement of tumor progression (Wu et al., 2017; Xu et al., 2018). Currently, exosome contents mirror the components of the host cells. Multiple types of proteins (receptors, enzymes, and transcript factors), lipids, and nucleic acids (DNA, mRNA, and ncRNA) inside and on the surface of the exosomes comprise their contents. Analysis on exosome protein composition revealed that adhesion molecules such as CAMs, integrins, tetraspanins, MHC class I/II, and transferrin receptors (TfR) are common among all exosomes, while the other proteins like Rab2, Rab7, flotillin, and annexin; heat shock proteins such as Hsp70 and Hsp90; and cytoskeleton proteins are non-specific proteins among exosomes (Mashouri et al., 2019).

### Roles of Exosomes in Tumor Biology

Tumor initiation and development are long-term and complex processes, which are prone to the influence of many factors. Hypoxic tumor microenvironment (TME) facilitates tumor progression, invasion, metastasis, and drug resistance. Exosomes released from the hypoxic TME contribute to these outcomes by transferring crucial signaling factors (Figure 2B). For instance, hypoxia tumor cells secret biomolecule-loaded exosomes that are highly enriched in immunomodulatory factors, chemokines, and oncoproteins, which finally enhance tumor progression (Park et al., 2019). Under hypoxic conditions, exosomes from ascites ovarian cancer cell lines carry the oncoproteins STAT3 and FAS and significantly increase cell invasion, chemoresistance, and metastasis (Dorayappan et al., 2018).

### Roles of Exosomes in Immune Regulation

Increasing evidence suggests the transportation of exosomes from tumor cells to immune cells, including Treg cells, NK cells, and macrophages, all of which have been shown to involve in the establishment and modulation of immunosuppressive microenvironment (Figure 2B). In this protumorigenic immunoenvironment, tumor cells escape the immune response and finally ensure their progression and metastasis (Zheng H. et al., 2018). Studies have demonstrated the presence of programmed cell death ligand-1 (PD-L1) in exosomes isolated from the plasma of lung cancer (LC) patients. Exosomal PD-L1 contributes to immunosuppression by reducing T-cell activity and promoting tumor growth (Chen G. et al., 2018; Kim et al., 2019). Moreover, it has been shown that exosomes internalized by NK cells lead to the proliferation and cytotoxicity of NK cells. For example, exosomal NF-κB-activating kinase-associated protein 1 (NAP1) (Wang et al., 2018) and IL15 (Borrelli et al., 2018) transferred to NK cells cause the enhancement of NK cells. Furthermore, tumor-derived exosomes induce polarization of macrophages, to either M1 or M2 phenotype. Oral squamous cell carcinoma (OSCC)-derived exosomes encapsulate miR-29a-3p to mediate M2 subtype polarization of macrophages and promote the proliferation and invasion of OSCC cells (Cai et al., 2019); human papillomavirus (HPV+) head and neck squamous cell carcinoma (HNSCC) transports exosomal miR-9 to macrophages and induces the M1 phenotype of macrophages, thereby increasing the radiosensitivity of HNSCC (Tong et al., 2020).

While tumor-derived exosomes regulate the phenotype and function of immune cells that can be beneficial for tumor evasion and immune destruction, immunocyte-released exosomes regulate the anti-tumor immune response, influencing various biological functions of both host and recipient cells. For instance, NK cell-derived exosomes mediate the cytotoxicity effect and inhibit tumor growth (Zhu et al., 2017; Neviani et al., 2019). M1 macrophage-derived exosomes transferring miR-16-5p to gastric cancer cells triggers T cell immune response by reducing the expression of PD-L1, which inhibits gastric cancer (GC) progression (Li et al., 2020). Overall, tumors employ diverse strategies to enhance development and evade immune responses, and exosomes are important mediators modulating tumor progression and metastasis by signal transportation and immune regulation within the TME.

## Molding of the Microenvironment by Exosomal ncRNAs

**FIGURE 3:**
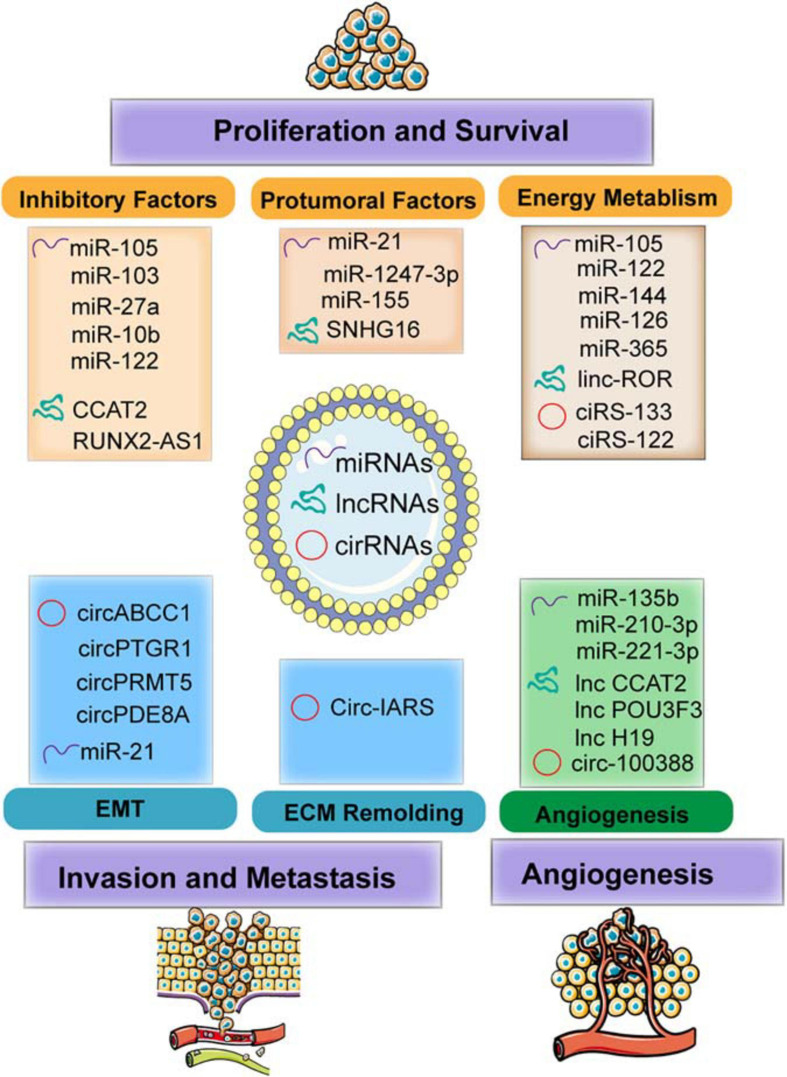
The functions of exosomal non-coding RNAs (ncRNAs) including microRNAs (miRNAs), long non-coding RNAs (lncRNAs), and circular RNAs (circRNAs) modulate the TME in facilitating the tumor progression and metastasis. Specifically, the mechanism involved in molding the TME including regulating energy metabolism (such as glycolysis and oxygen metabolism) and transmitting the protumoral or inhibitory factors to recipient cells. In addition, exosomes transferred to endothelial cells (ECs) induce angiogenesis by regulating the features of ECs. Furthermore, exosomes also mediated invasion and metastasis by epithelial–mesenchymal transition (EMT) and extracellular matrix (ECM) remodeling.

This chapter focused on the different types of exosomal ncRNAs (including miRNA, lncRNA, and circRNA)-based regulation in the microenvironment modulation through different mechanisms, including energy metabolism regulation, protumor signal transportation, and inhibitory factor blockage, resulting in tumor cell growth and survival, inducing angiogenesis, and enhancing ECM remodeling and EMT process, thereby contributing to tumor invasion and metastasis (Figure 3 and Table 1).

**TABLE 1:** Roles of tumor-derived exosomal ncRNAs in modulating the TME.

ncRNAs	Host cell	Target/Mechanism	Effect	Function	References
miR-105	Breast cancer	Myc	Glycolysis;Glutamine decomposition	Proliferation	Yan et al., 2018
miR-122	Breast cancer	Glycolytic pyruvate kinase	Energy metabolism	Progression	Basu and Bhattacharyya, 2014
	Hepatoma cells	IGF-1	IGF1-dependent/miR-122 signal	Growth	Fong et al., 2015
miR-144	Breast Cancer	MAP3K8/ERK1/2/PPARγ pathway	Autophagy/Adipocytes catabolism	Progression	Wu et al., 2019a
miR-126		AMPK/HIF1α IRS/Glut-4 signaling			
miR-21	Hepatocellular carcinoma	PTEN/PDK1/AKT signaling	Convert HSCs to CAFs	Progression	Zhou Y. et al., 2018
miR-1247-3p	Hepatocellular Carcinoma	B4GALT3/β1-integrin/NF-κB signaling	IL-6/IL-8	Progression	Fang T. et al., 2018
miR-155	Arsenite-transformed Hepatic Epithelial Cells	NF-κB	IL-6/IL-8	Carcinogenesis	Chen et al., 2017
miR-105	Breast Cancer	ZO-1	Endothelial junction/Vascular permeability	Metastasis	Zhou et al., 2014
miR-103	Hepatoma cell	VE-cadherin/p120/ZO-1	Vascular permeability	Metastasis	Fang J. H. et al., 2018
miR-27a	Prostate Cancer	P53	/	Chemoresistance	Cao et al., 2019
miR-10b	Colorectal Cancer	PIK3CA/PI3K/Akt/mTOR pathway	TGF-β/SM α-actin	Growth	Dai et al., 2018
miR-135b	Myeloma cells	FIH-1/HIF signaling	Endothelial tube formation	Angiogenesis	Umezu et al., 2014
miR-210-3p	Hepatocellular Carcinoma	SMAD4/STAT6	HUVECs tubulogenesis	Angiogenesis	Lin et al., 2018
miR-221-3p	Cervical Squamous cell Carcinoma	VASH1/ERK/AKT signaling	HLECs migration/Tube formation	Lymphangiogenesis/Lymphatic metastasis	Zhou et al., 2019
miR-21	Oral Squamous Cell Carcinoma	Snail/vimentin/E-cadherin	EMT	Metastasis	Li et al., 2016
lncRNA ROR	Hepatocellular Carcinoma	miRNA-145/HIF-1α/PDK1	Glycolysis	Growth and Survival	Takahashi et al., 2014
SNHG16	Breast Cancer	CD73/miR-16-5p	TGF-β1/SMAD5 pathway	Immunosuppression	Ni et al., 2020
RUNX2-AS1	Myeloma	RUNX2	Splicing efficiency	Osteogenesis	Li B. et al., 2018
CCAT2	Glioma	Bcl-2/Bax; VEGFA/TGFβ	HUVEC apoptosis	Angiogenesis	Lang et al., 2017b
POU3F3	Glioma	bFGF/bFGFR/VEGFA	HBMECs tubular-like structure formation	Angiogenesis	Lang et al., 2017a
H19	CD90+ liver cancer	VEGFR1	Tubular-like structures	Angiogenesis	Conigliaro et al., 2015)
ciRS-133	Gastric Cancer	PRDM16/UCP1	Oxygen consumption/Heat production	Tumor cachexia	Zhang et al., 2019b
ciRS-122	Colorectal Cancer	miR−122/PKM2	Glycolysis	Chemoresistance	Wang et al., 2020
circRNA 100388	Hepatocellular Carcinoma	/	Vasculogenic formation	Angiogenesis/Invasion/Metastasis	Huang et al., 2020
Circ-IARS	Pancreatic Cancer	miR-122/ZO-1/RhoA	RhoA-GTP/Focal adhesions/Endothelial monolayer permeability	Invasion and Metastasis	Li J. et al., 2018
Circ-PTGR1	Hepatocellular Carcinoma	miR449a-MET	TME homeostasis destruction	Development	Wang et al., 2019
Circ-ABCC1	Colorectal Cancer	β-catenin/Wnt/β-catenin pathway	Stemness/Sphere formation	Metastasis	Zhao et al., 2020
Circ-PRMT5	Bladder Cancer	miR-30c/SNAIL1/E-cadherin pathway	Epithelial-Mesenchymal Transition	Progression and Metastasis	Chen X. et al., 2018
Circ-PDE8A	Pancreatic Ductal Adenocarcinoma	miR-338/MACC1/MET	Invasive growth	Invasion and Progression	Li Z. et al., 2018

### Exosomal miRNAs Modulate TME to Facilitate Tumor Progression

#### Energy Metabolism

Glucose metabolism reprogramming is considered as a hallmark of cancer. For example, breast cancer-derived exosome-encapsulated miR-105 mediates metabolic reprogramming of stromal cells and activates the Myc signaling in the CAFs, thereby contributing to tumor proliferation (Yan et al., 2018). In detail, miR-105 transferred to CAF can increase glycolysis and glutamine decomposition levels and release acetic acid and glutamate into the TME to provide energy or raw materials for tumor or protein synthesis. Surprisingly, when the energy is insufficient, miR-105 can initiate gluconeogenesis and detoxification, converting the harmful metabolites lactic acid and ammonia into acetic acid and amino acids, which can be released into the TME for cancer cell. Moreover, cancer cell-derived miR-122 increases the utilization of nutrients in the pre-metastatic niche and decreases glucose uptake of niche cells by inhibiting glycolytic pyruvate kinase, leading to energy metabolism reprogramming to facilitate tumor progression (Fong et al., 2015).

In addition to glucose metabolism, the contents transferred by exosomes also exhibit important roles in the TME through fatty acid metabolism. For instance, adipocytes co-cultured with exosomes derived from tumor cells are in an activated catabolic state and release a large number of metabolites, including free fatty acids, pyruvate, lactic acid, and ketone bodies. Tumor cells activate the beige-brown phenotype differentiation in adipocytes by encapsulating high levels of miRNA-144 and miRNA-126 via exosomes, enhance the catabolism of recipient adipocytes, and ultimately promote tumor progression through metabolic reprogramming (Wu et al., 2019a). Specifically, exosomal miRNA-144 promotes beige-brown adipocyte characteristics by down-regulating the MAP3K8/ERK1/2/PPARγ pathway, while exosomal miRNA-126 activates the AMPK-related autophagy pathways and promotes the expression of HIF1α to achieve metabolic regulation by inhibiting IRS/Glut-4 signaling. Tumor-derived exosomes serve as fatty acid carriers, inducing the conversion of metabolism to mitochondrial oxidative phosphorylation (Yin X. et al., 2020). In turn, adipocytes delivered fatty acids and enzymes involved in fatty acid oxidation to cancer cells through EVs, which promote fatty acid oxidation and melanoma cell migration (Clement et al., 2020).

Immune cells and cancer cells also release miRNAs to regulate nucleic acid metabolism. For example, TAM releases miR-365-rich exosomes that are uptaken by pancreatic cancer cells and induce gemcitabine (cytidine analog) resistance through promoting pyrimidine metabolism, thereby increasing the nucleotide triphosphate pool that competes with gemcitabine to enter the DNA of cancer cells. In addition, miR-365 also upregulates the cytidine deaminase (CDA) level, which catabolizes gemcitabine (Binenbaum et al., 2018).

#### Decreasing the Inhibitory Factors

As major components in the exosomes, miRNAs in exosomes released from metastatic cells are shown to be of higher enrichments compared to those from non-metastatic cells (Melo et al., 2014). Exosomal miRNAs are capable of molding the TME by restraining the inhibitory genes or proteins, thereby relieving the negative effects and facilitating tumor growth. For example, exosome-mediated transfer of miR-105 and miR-103 efficiently destroys the endothelial junctions and the integrity of these natural barriers against metastasis. The overexpression of miR-105 in non-metastatic cancer cells induces metastasis and vascular permeability by targeting junction proteins (Zhou et al., 2014), while miR-103 directly inhibits the expression of VE-cadherin (VE-Cad), p120-catenin (p120), and zonula occludens 1 (ZO-1) (Fang J. H. et al., 2018). Moreover, exosomes positively enhance tumor progression by modulating stromal cells within the TME. For example, exosome-derived miR-27a is involved in chemoresistance of prostate cancer (PCa) cells. After co-culturing PCa cells (PC3 cells) with primary prostate fibroblasts (PSC27 cells), exosomal miR-27a released from PSC27 cells finally improves chemoresistance by inhibiting the expression of P53 (Cao et al., 2019). In addition, miR-10b released from colorectal cancer (CRC) cells is transferred to fibroblasts and reduces fibroblast proliferation, but promotes the expression of TGF-β and SM α-actin; it also restrains the PIK3CA expression levels and decreases the activity of the PI3K/Akt/mTOR pathway. All these results suggest that exosomal miR-10b may activate the fibroblasts to facilitate CRC growth (Dai et al., 2018). Furthermore, the expression of exosomal miR-122 released by hepatocellular carcinoma (HCC) Huh7 cells is restrained by insulin-like growth factor 1 (IGF-1) secreted from the recipient HCC HepG2 cells, which can also reduce the expression of miR-122 in Huh7 cells, thereby ensuring HepG2 cell growth (Basu and Bhattacharyya, 2014).

#### Increasing Protumor Signals

Exosomal miRNAs modulate the TME through transmitting protumor signals to recipient cells. HCC cell-derived exosomal miRNA-21 induces the activation of hepatic stellate cells (HSCs), which can transform normal HSCs into CAFs (Zhou Y. et al., 2018). It is also worth noticing that miR-1247-3p secreted by HCC cells and carried via exosomes induces fibroblast activation to foster metastatic niche (Fang T. et al., 2018). Those activated CAFs further promote HCC development by secreting multiple angiogenic cytokines. Mechanically, miR-21 directly targets PTEN and activates the pyruvate dehydrogenase kinase isoenzyme 1 (PDK1)/AKT signaling pathway (Zhou Y. et al., 2018), whereas miR-1247-3p straightly targets B4GALT3, activating the β1-integrin/NF-κB signaling in fibroblasts (Fang T. et al., 2018). Moreover, exosomal miRNAs are involved in regulating inflammatory infiltration intrigued by environmental chemical-induced carcinogenesis. It has been demonstrated that exosomal miR-155 is upregulated in the serum of arsenite-exposed individuals and arsenite-transformed cells. The arsenite-transformed cells transfer miR-155 to recipient cells through exosomes to induce the pro-inflammatory activity of normal hepatocytes (Chen et al., 2017). Thus, the interaction between the recipient cell and the host cell through the transfer of exosomal miRNAs indicates that the tumor cells modulate its microenvironment by transmitting regulatory factors to facilitate their growth and survival.

#### Inducing Angiogenesis

Exosome-mediated transfer of miRNAs also modulates the biology of endothelial cells (ECs) and contributes to tumor angiogenesis. For example, exosomal miR-210-3p derived from multiple types of HCC cell lines can be delivered into ECs and then promote tubulogenesis of human umbilical vein endothelial cells (HUVECs) by inhibiting the SMAD4 and STAT6 levels, thereby facilitating tumor angiogenesis (Lin et al., 2018). With the knowledge that tumor angiogenesis is also initiated under hypoxic environment, exosomes derived from glioblastoma cells under hypoxic conditions, compared with normoxic conditions, definitely cause a stronger increase in tumor vasculature and cell proliferation (Zhang G. et al., 2017). Furthermore, hypoxic cancer cells may activate hypoxic signals and release more exosomes to their microenvironment to promote their own invasive phenotypes (King et al., 2012). In addition, exosomes from hypoxia-resistant multiple myeloma (HR-MM) cells are significantly enriched of miR-135b, which enhances the endothelial tube formation via the HIF signaling (Umezu et al., 2014). Circulating exosomal miR-221-3p from CSCC to human lymphatic endothelial cells (HLECs) results in the increased migration and tube formation of HLECs, and facilitates lymphangiogenesis through targeting VASH1 and activating the ERK/AKT signaling (Zhou et al., 2019). Thus, these exosomal miRNAs constitute potential drivers of hypoxia-dependent angiogenesis and contact with surrounding environment during tumor progression.

#### Promoting ECM Remodeling and EMT Process

Cancer cell-released exosomes would activate the epithelial–mesenchymal transition (EMT) and induce extracellular matrix (ECM) remodeling via secreting active, invasive-related soluble factors to modulate the local and distant TME for the implementation of tumor invasion and metastasis. For example, cancer cell-derived miR-122 increases the utilization of nutrients in the pre-metastatic niche and decreases glucose uptake by niche cells by inhibiting glycolytic pyruvate kinase, leading to energy metabolism reprogram to facilitate tumor progression (Fong et al., 2015). It is also shown that tumor cells generate miR-21-enriched exosomes under hypoxic microenvironment. Exosomal miR-21 is transferred to normoxic cells, significantly promotes snail and vimentin expression, and reduces the levels of E-cadherin, contributing to metastasis-promoting behaviors (Li et al., 2016).

### Exosomal lncRNAs Modulate TME to Facilitate Tumor Progression

#### Regulating Energy Metabolism

Tumor cells reprogram energy metabolism and achieve malignant phenotypes under hypoxic conditions. High levels of linc-ROR-encapsulated exosomes secreted by hypoxic HCC promote the glycolysis process and oppose a hypoxic environment. Exosomal linc-ROR significantly increases the expression of miRNA-145, HIF-1α, and pyruvate dehydrogenase kinase isoenzyme 1 (PDK1), thereby promoting cell growth and survival (Takahashi et al., 2014).

#### Transmitting Protumor and Inhibitory Signals

The transmission of the crucial signal factors between cancer cells and stromal cells via exosomes becomes the driving factor of tumor development. The decreased osteogenic potential of mesenchymal stem cells (MSCs) is one of the main features of malignant mesothelioma (MM). lncRNA RUNX2-AS1 is enriched in MSCs from MM patients, and it can be packaged into exosomes derived from myeloma cells and transferred to MSCs and then transcriptionally inhibit the expression of RUNX2 by reducing its splicing efficiency, thereby decreasing the osteogenic effects of MSCs (Li B. et al., 2018). It has also been shown that lncRNA CCAT2 is overexpressed in glioma tissues and ncU87 cell-released exosomes. ncU87-released exosomes are enriched in CCAT2, which are transferred to HUVEC and then increase the levels of Bcl-2 and inhibit the expression of Bax and caspase-3, thus decreasing HUVEC apoptosis induced by hypoxia (Lang et al., 2017b). In addition, lncRNAs encapsuled by exosomes are considered as important mediators for the phenotype of immune cells. For example, exosomes derived from breast cancer cells can deliver SNHG16 to Vδ1T cells and upregulate the expression of CD73 in Vδ1T cells. In terms of mechanism, SNHG16 acts as a ceRNA by sponging miR-16-5p, downregulating its target gene SMAD5 and activating the TGF-β1/SMAD5 pathway, thereby converting γδ1 T cells in to the CD73+-infiltrating T cells, which display immunosuppressive phenotype in the TME (Ni et al., 2020). Thus, these results prove the importance of exosomes in the TME and that targeting these lncRNAs or blocking the transfer of exosomes might have potential therapeutic effects for cancer in the future.

#### Angiogenesis

The exosome-mediated crosstalk between tumor cells and endothelial cells could facilitate angiogenesis through transfer of lncRNAs (Lang et al., 2017a, b). Exosomal linc-CCAT2 released from ncU87 is uptaken by HUVEC. The overexpression of CCAT2 in HUVECs results in the activation of VEGFA and TGFβ, thereby promoting angiogenesis of glioma cells (Lang et al., 2017b). Moreover, glioma A172 cells can also induce angiogenesis by secreting exosomes containing linc-POU3F3 and upregulate the expression level of angiogenesis-related genes such as bFGF, bFGFR, and VEGFA in human brain microvascular endothelial cells (HBMECs) (Lang et al., 2017a). These exosomal lncRNAs finally enhance the migration and proliferation of ECs and the structure and small artery formation of tubules. Moreover, exosomes released by CD90+ aggressive cancer stem cell (CSC)-like cancer cells also modulate the TME by promoting angiogenic phenotype and cell adhesion. Studies have demonstrated that HUVECs internalize diverse exosome cargoes from multiple cell types. Co-culture of HUVECs with the CD90+ exosomes increases the expression of VEGF and the adhesion molecule ICAM-1, as well as enhances tubular-like structures. Thus, the transfer of H19 lncRNA from CD90+ cells to HUVECs through exosomes affects the levels of VEGFR1, suggesting a potency of lncRNA H19 in favor of angiogenesis (Conigliaro et al., 2015).

### Exosomal circRNAs Modulate TME to Facilitate Tumor Progression

#### Endothelial Permeability

Like other ncRNAs, circRNAs can be carried from cancer cells to other cells surrounding the microenvironment through exosomes, which act as crucial messengers to mediate intracellular communication. It has been confirmed that the expression of circ-IARS is positively correlated with liver metastasis, vascular infiltration, and TNM stages and negatively correlated with postoperative survival. In addition, circ-IRAS can also access HUVEC through exosomes derived from pancreatic cancer cells, significantly decreasing the levels of miR-122 and ZO-1; increasing the levels of RhoA, RhoA-GTP, and focal adhesions; and subsequently increasing the permeability of the endothelial monolayer and facilitating tumor invasion and metastasis (Li J. et al., 2018). In addition, the transfer of exosomal circRNA-100388 from HCCs to HUVECs leads to the induction of proangiogenic activity and regulates angiogenesis (Huang et al., 2020). This finding indicates that the existence of circRNAs in exosomes may be an important mediator involved in intercellular communication, as well as potential biomarkers for tumor diagnosis and prognosis.

#### EMT and Metastasis

Cancer cells with higher metastasis confer potential functions on those with lower or no metastatic potential via exosomes, resulting in the enhancement of migration and invasive abilities of cancer cells. For instance, circPTGR1 is specifically expressed in exosomes from low metastatic (97L) and high metastatic (LM3) cells and also upregulated in serum exosomes from HCC patients and associated with clinical staging and prognosis. The enhanced migration and invasion of HepG2 and 97L cells induced by co-culturing with LM3 exosomes can be reversed after knockdown of circPTGR1 expression. Furthermore, exosomes derived from highly metastatic cells release circPTGR1, which affects the metastatic potential of recipient cells by targeting the miR449a–MET interaction, leading to the destruction of the TME homeostasis and facilitating HCC development (Wang et al., 2019). In addition, exosomal circRNAs such as hsa_circ_0000677 (circ-ABCC1) enhances CRC cells stemness and sphere formation and contributes to tumor invasion and progression (Zhao et al., 2020). Of note is the exosomal communication between tumor cells that identifies the tumor-released exosomes in the circulation and that high levels of circPRMT5 and circ-PDE8A in plasma exosomes of tumor patients are associated with tumor progression and prognosis. Studies reveal that circPRMT5 in the serum and urine exosomes of urothelial carcinoma of the bladder tissue enhances the EMT process via sponging miR-30c through the SNAIL1/E-cadherin pathway (Chen X. et al., 2018), whereas circ-PDE8A from liver-metastatic pancreatic ductal adenocarcinoma (PDAC) cells acts as a ceRNA of miR-338 to upregulate MACC1 and MET to increase the invasive growth of PDAC cells, thereby affecting tumor progression and metastasis (Li Z. et al., 2018). Therefore, numerous studies suggest that exosome-derived circRNAs can mediate the invasive phenotype between tumor and TME and are involved in multiple biological processes of different types of tumors.

#### Energy Metabolism

Cancer-secreted circRNAs in the plasma exosomes are associated with WAT browning and metabolic dysfunction. Exosomes from gastric cancer (GC) cells deliver ciRS-133 to adipocytes, which inhibits the expression of miR-133 and activates the expression of PRDM16 and UCP1, thereby accelerating the metabolic rate of mature adipocytes, increasing oxygen consumption and heat production. This circRNA ultimately aggravates tumor cachexia (Zhang et al., 2019b). Moreover, malignant solid tumors metabolize glucose through aerobic glycolysis to generate lactate and ATP, achieving rapid tumor growth and chemotherapy resistance. Exosomes from oxaliplatin-resistant cells deliver circ_0005963 (termed ciRS-122) to sensitive cells, promoting glycolysis and drug resistance as a miR-122 sponge and enhancing the expression of PKM2. In addition, *in vivo* experiments reveal that decreasing si-ciRS-122 transportation could suppress glycolysis and increase sensitivity to oxaliplatin (Wang et al., 2020). Thus, these *in vitro* and *in vivo* experiments suggest that targeting exosome cargoes regulating energy metabolism, including lipid metabolism and glycolysis, would provide a novel and effective way for cancer therapy.

## Regulation of Tumors by Stromal Cell-Derived Exosomal ncRNAs

**FIGURE 4:**
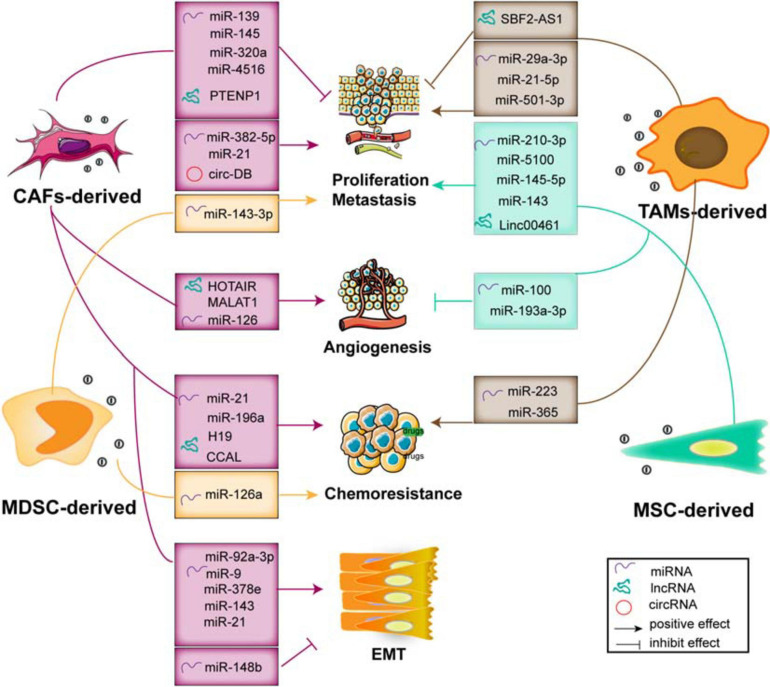
The functions of exosomal ncRNAs derived from stromal cells within the TME contribute to tumor progression and metastasis, angiogenesis, EMT, and chemoresistance.

The stromal cells within the TME include CAFs, multiple types of immune cells (such as Treg cells, MSCs, and macrophages), and bone marrow-derived precursor cells, which dynamically interregulate with cancer cells surrounding the TME (Figure 4 and Table 2). As we have known, these types of cells not only regulate tumor growth and maintenance but also modulate responses and biological functions of immune cells (Nabet et al., 2017). Exosomal RNAs, which are largely non-coding transcripts and transposable elements and transferred from stroma to tumor cells, have been implicated in all stages of tumor progression and chemotherapeutic resistance (Boelens et al., 2014). Based on the genetic stability of stromal cells, they represent a class of attractive therapeutic targets within the TME (Luga et al., 2012).

**TABLE 2:** Roles of stromal cell-derived exosomal ncRNAs in tumor biology.

Host cell	ncRNAs	Target/Mechanism	Effect	Function	References
CAFs	miR-139	MMP11	/	Growth and Metastasis	Xu et al., 2019
	miR-145	/	Apoptosis	Growth	Han et al., 2018
	miR-382-5p	/	/	Progression	Sun et al., 2019
	miR-21	/	Liver Metastasis	Migration and Invasion	Bhome et al., 2017
		APAF1	Apoptosis	Chemoresistance	Au Yeung et al., 2016
	miR-320a	PBX3/MAPK pathway	CDK2/MMP2/EMT	Proliferation/Migration/Metastasis	Zhang Z. et al., 2017
	miR-148b	DNMT1	EMT	Progression	Li et al., 2019
	miR-4516	FOSL1	/	Proliferation	Kim et al., 2020
	miR-126	VEGF/EGFL7/IRS1	ECs tube formation	Angiogenesis and Proliferation	Monaco et al., 2019
	miR-9	E-cadherin	ECM remodeling	Growth	Baroni et al., 2016
	miR-21/miR-378e/miR-143	EMT markers	Stemness	Growth and Invasion	Donnarumma et al., 2017
	miR-92a-3p	FBXW7/MOAP1/CD133/CD44/OCT4/N-cadherin/vimentin	Stemness/EMT	Metastasis and Chemoresistance	Hu et al., 2019
	miR-196a	hnRNPA1/CDKN1B/ING5	/	Growth and Chemoresistance	Qin et al., 2019
	LncRNA PTENP1	PTEN/miR-17	Apoptosis	Invasion and Migration	Zheng R. et al., 2018
	H19	miR-141	Stemness	Development and Chemoresistance	Ren et al., 2018
	CCAL	HuR/β-catenin	mRNA stabilization/apoptosis	Development and Chemoresistance	Deng et al., 2020
	HOTAIR/MALAT1	/	pro-angiogenic effects	Angiogenesis	Lamichhane et al., 2017
	circ-DB	miR-34a/USP7/cyclin A2 signaling	DNA damage/Deubiquitination	Development	Zhang et al., 2019a
MSCs	miR-145-5p	Smad3	Apoptosis	Proliferation and Metastasis	Ding et al., 2019
	miR-143	TFF3/PCNA/MMP2/9	Apoptosis	Proliferation Migration and Invasion	Che et al., 2019
	miR-100	VEGF/mTOR/HIF-1α signaling	ECs vascular behavior	Angiogenesis	Pakravan et al., 2017
	miR-193a-3p/miR-210-3p/miR-5100	Mesenchymal related molecules/STAT3 signaling	/	Metastasis	Zhang et al., 2019b
	linc00461	miR-15a/16/Bcl-2	Apoptosis	Growth	Deng et al., 2019
MDSC	miR-143-3p	ITM2B/PI3K/Akt signaling	/	proliferation	Zhou et al., 2020
	miR-126a	IL-13R/S100A8/9	Th2 cell response/Tube formation	Angiogenesis	Deng et al., 2017
TAM	miR-365	Cytidine Deaminase	Pyrimidine Metabolism	Chemoresistance	Binenbaum et al., 2018
	miR-501-3p	TGFBR3/TGF-β signaling	/	Migration and Invasion	Yin et al., 2019
	miR-223	PTEN/PI3K/AKT pathway	/	Chemoresistance	Zhu X. et al., 2019
	SBF2-AS1	miR-122-5p/XIAP	Tumorigenic ability	Progression	Yin Z. et al., 2020
	miR-29a-3p/miR-21-5p	STAT3	Treg/Th17 ratio	Progression and Metastasis	Zhou Y. et al., 2018

### CAF-Derived Exosomes Promote Tumor Development and Progression

Fibroblasts, specifically the CAFs, are the main type of non-immune cells within the TME (Denton et al., 2018). Much evidence suggests that many ncRNAs including miRNAs (Bhome et al., 2017; Qin et al., 2019; Sun et al., 2019), lncRNAs (Ren et al., 2018; Zheng R. et al., 2018; Deng et al., 2020), and circRNAs (Zhang et al., 2019a) are overexpressed in CAF-derived exosomes, and these ncRNAs influence the growth, invasion, and chemoresistance in recipient tumor cells via regulation of multiple signaling pathways. Herein, we summarize the crucial biological and pathogenic roles of ncRNAs released from CAF exosomes in tumors.

#### Exosomal miRNAs

##### Proliferation and Metastasis

Multiple evidence shows that many exosomal miRNAs derived from CAFs regulate the proliferation and invasion of cancer cells (Savardashtaki et al., 2019). Exosomes effectively deliver exogenous miRNAs to cancer cells, inserting positive or negative effects on cell proliferation and invasion. For example, studies demonstrate that exosomal miR-139 derived from CAFs (Xu et al., 2019) and miR-145 secreted by stroma cell exosomes (Han et al., 2018), respectively, suppress GC and PDAC cell proliferation and invasion. Otherwise, CAF-derived exosomes transport miR-382-5p (Sun et al., 2019) and miR-21 to recipient OSCC and CRC cells, leading to the enhancement of cancer cell migration and invasion. Compared with the orthotopic xenotransplantation constructed with fibroblasts, the xenograft-transplanted tumor, which is constructed with fibroblasts stably overexpressing miR-21, exhibits a significantly increased level of liver metastasis (Bhome et al., 2017).

Although the exosomes carry enriched miRNAs to cancer cells and influence tumor proliferation and metastasis, the loss of exosomal miRNAs from CAFs has a profound and positive impact on tumor progression. The levels of exosomal miR-320a, miR-148b, and miR-4516 derived from CAFs are significantly decreased, and they function as anticancer miRNAs. In detail, miR-320a inhibits HCC cell proliferation, migration, and metastasis by targeting the PBX3/MAPK pathway (Zhang Z. et al., 2017); miR-148b suppresses EMT and endometrial cancer metastasis by binding to DNMT1 (Li et al., 2019); and miR-4516 is negatively associated with the expression of FOSL1 and inhibits breast cancer cell malignancy and proliferation (Kim et al., 2020). Thus, these results indicate that CAF-mediated cancer progression and metastasis are partially related to the loss of miRNA expression in CAF-derived exosomes, resulting in the alleviation of their target genes inhibition, and the stromal cell-derived miRNAs may be potential factors to prevent tumor development.

##### Angiogenesis

It has been demonstrated that the interacted communication between malignant mesothelioma (MM) cells and matrix components regulates the distribution of miR-126 in the microenvironment, thereby modulating angiogenesis and cell proliferation (Monaco et al., 2019). After transferring miR-126 via EC-derived exosomes, the reduction of miR-126 content in fibroblasts is beneficial for ECs to inhibit angiogenesis and cell growth. Conversely, the accumulation of miR-126 in fibroblasts and the reduction of miR-126 levels in ECs enhance tube formation through upregulation of VEGF/EGFL7/IRS1.

##### Stemness and EMT

Tumor cells co-cultured with CAF exosomes or transfected with those miRNAs derived from CAF exosomes exhibit an increased capacity of mammosphere potency and EMT process. Tumor-derived exosomal miR-9 is delivered to normal fibroblasts (NFs) and enhances cell motility and, in turn, it also can be released from NFs to breast cancer cells. Co-culture with triple negative breast cancer (TNBC) cells and conditioned medium derived from miR-9 overexpressing NFs promotes the cancer cell migration by modulating the expression of E-cadherin and enhances the NF migration and invasion, resulting in ECM remodeling and tumor progression (Baroni et al., 2016). Of note is that, compared with normal fibroblasts, the expression of miR-21, miR-378e, miR-143 (Donnarumma et al., 2017), and miR-92a-3p (Hu et al., 2019) in the CAF exosomes is significantly increased. In detail, CAF-secreted exosomes mediate the transmission of miR-21, miR-378e, and miR-143 into breast cancer T47D cells, which causes a significant enhancement in the number and diameter of mammospheres and increased expression of stemness and EMT markers, thereby promoting aggressive growth and invasion of cancer cells (Donnarumma et al., 2017). Moreover, CAFs transfer exosomal miR-92a-3p to CRC cells and facilitate cell stemness, EMT, metastasis, and 5-FU/L-OHP resistance by directly targeting FBXW7 and MOAP1 (Hu et al., 2019); it also increases sphere formation and the levels of stemness markers CD133, CD44, and OCT4; enhances the expression of N-cadherin and vimentin; and suppresses mitochondrial apoptosis in CRC. Therefore, these findings suggest that inhibiting the transfer of exo-miRNAs would become an alternative measure in the treatment of cancer metastasis and chemoresistance.

##### Chemoresistance

The malignant phenotype and chemosensitivity of cancer cells can be altered by the miRNA transfer via exosomes derived from stromal cells within the TME. It has been found that the levels of miRNA-21 in the exosomes and tissue lysates derived from cancer-associated adipocytes (CAAs) and CAFs are significantly higher than those in ovarian cancer cells (Au Yeung et al., 2016), which can be transferred from both CAAs and CAFs to ovarian cancer cells via exosomes, where it exerts no influence on cell growth but induces cell invasion, suppresses cancer apoptosis by directly targeting APAF1, and contributes to paclitaxel chemoresistance. In addition, CAF-derived exosomal miR-196a can be uptaken by head and neck cancer (HNC) cells and enhances cell growth and cisplatin resistance, which may be mediated by the translocation of heterogeneous nuclear ribonucleoprotein A1 (hnRNPA1) and directly downregulate the expression of CDKN1B and ING5 (Qin et al., 2019). These findings indicate that CAF-released exosomal miRNAs are transferred from fibroblasts to different cancer cells, which mainly exert their biological effects through direct binding with their downstream targets. However, the upstream mechanism still needs further exploration and confirmation.

#### Exosomal lncRNAs

Multiple studies have demonstrated that lncRNAs mediate cell-to-cell communication within the TME via exosomes, leading to the progression and chemoresistance of cancer cells. For example, exosomal PTENP1 suppresses the malignant phenotype of BC cells, which acts as a ceRNA sponge to promote the expression of PTEN by inhibiting the miR-17 levels, thereby inducing apoptosis and inhibiting BC cell invasion and migration (Zheng R. et al., 2018). In addition, CAF-derived exosomes participate in the upregulation of H19 (Ren et al., 2018) and colorectal cancer-associated lncRNA (CCAL) (Deng et al., 2020) and enhance CRC cell development and chemoresistance through activating the β-catenin pathway. The transfer of CAF-derived exosomal H19 acts as a ceRNA sponge for miR-141, which significantly suppresses the stemness of CRC cells, while exosomal CCAL straightly binds with human antigen R (HuR) and facilitates oxaliplatin resistance of recipient CRC cells. Otherwise, it has been shown that the upregulation of HOTAIR and MALAT1 in endothelial EVs induced by ethanol mediates the pro-angiogenic effects (Lamichhane et al., 2017). Therefore, it is worth exploring the novel lncRNAs enriched in the exosomes derived from endothelial cells surrounding the TME, which may play a crucial role in secreting certain vascular growth factors, promoting pro-angiogenesis phenotypes, and contributing to tumor progression.

#### Exosomal circRNAs

circRNAs have recently been considered as key factors in modulating tumor development. It has been shown that circRNAs derived from adipose tissue certainly regulate deubiquitination and promote HCC cell growth. Circ-deubiquitination (circ-DB) encapsuled in adipose exosomes from HCC patients is upregulated and inhibits DNA damage by modulating the deubiquitination-related miR-34a/USP7/cyclin A2 signaling pathway (Zhang et al., 2019a). Therefore, the exploration on the novel circRNAs carried by fibroblast-derived exosomes can provide a novel insight for cancer therapy, so further studies are needed to uncover the different regulatory mechanisms that modulate the malignant phenotypes of tumors.

### MSC-Derived Exosomes Modulate Tumor Progression

Mesenchymal stem cells (MSCs) are vital components of the TME. They modulate tumorigenic and metastatic capacities, as well as stimulate EMT by direct communication with cancer cells via exosomes in tumor stroma. Multiple studies have shown that exosomes from MSCs deliver miR-145-5p and miR-143, which inhibit cancer cell proliferation and metastasis, as well as induce cell apoptosis (Ding et al., 2019). Mechanically, exosomal miR-145-5p shuttles the expression of Smad3 *in vitro*; transfer of exosome-miR-143 inhibits the expression of trefoil factor 3 (TFF3), proliferating cell nuclear antigen (PCNA), matrix metalloproteinase (MMP)-2, and MMP-9 (Che et al., 2019). In addition, the exosomal miR-100 from MSCs to BC cells is associated with the decreased expression of vascular endothelial growth factor (VEGF) and the activation of the mTOR/HIF-1α signaling, which suppresses the angiogenesis of ECs (Pakravan et al., 2017). In turn, exosomes derived from hypoxic BMSCs exert malignant behaviors of recipient cancer cells. The transfer of miR-193a-3p, miR-210-3p, and miR-5100 (Zhang X. et al., 2019) from BMSCs to epithelial cancer cells via exosomes results in the activation of the STAT3 signaling and the upregulation of the mesenchymal-related molecules, thereby facilitating invasion and metastasis of lung cancer cells. Furthermore, it has been demonstrated that linc00461 is highly enriched in MSC-derived exosomes. Exosomal linc00461 promotes multiple myeloma cell growth and induces cell apoptosis by inhibiting the miR-15a/16 levels and promoting the expression of Bcl-2 (Deng et al., 2019). Overall, as a novel type of ncRNAs, circRNAs play crucial parts in multiple biological processes during tumor initiation and progression. However, little is known about the function of circRNAs derived from MSC-derived exosomes in modulating proliferation and differentiation of multiple tumor cells.

### MDSC-Derived Exosomes Mediate the Immunosuppressive Functions

MDSCs are immature myeloid cells that accumulate in the internal circulation and mediate the immunosuppressive phenotype within the TME that suppresses adaptive and innate immunity and blocks immunotherapy. They release exosome cargoes, biologically functional multiple miRNAs that suppress the differentiation and activation (Guo et al., 2018) and increase the proliferation of myeloid cells (Chen et al., 2015; Geis-Asteggiante et al., 2018). For example, exosomal miR-143-3p released from granulocytic myeloid-derived suppressor cells (G-MDSCs) is increased in G-MDSC-derived exosomes. It decreases the integral membrane protein 2B (ITM2B) and activates the PI3K/Akt signaling pathway, thereby accelerating cell proliferation (Zhou et al., 2020). In addition, chronic inflammation plays a crucial role in acquired chemoresistance, which is partially due to the induction and expansion of MDSCs and IL-13+ Th2 cells. It has been shown that MDSC exosomes treated with doxorubicin (DOX) facilitate tumor angiogenesis and metastasis by the crosstalk between Th2 T cells and ECs through exosomal miR-126a. The expression of miR-126a in MDSC is induced by DOX and can be enveloped into MDSC exosomes, inducing Th2 cell response and tube formation. Interestingly, IL-13+ Th2 cells induced by DOX in turn promote the production of MDSC-miR-126a-enriched exosomes by binding with IL-13R. Noticeably, MDSC miR-126a enhances the accumulation of MDSCs and tumor angiogenesis through regulation of S100A8/9 (Deng et al., 2017). However, the effect of the contents of exosomes secreted by MDSCs and its function on tumor still need further study.

### TAM-Derived Exosomes Facilitates Tumor Invasion and T-Cell Regulation

As the main immune cells in the TME, TAMs contact with cancer cells through transferring exosome cargoes to regulate tumor proliferation, invasion, and angiogenesis. For instance, it has been demonstrated that miR-501-3p is highly enriched in TAM-derived exosomes, which decreases the TGFBR3 levels through the activation of the TGF-β signaling, thereby promoting PDAC cell migration and invasion (Yin et al., 2019). In addition, the delivery of exosomal miR-223 to the co-cultured hypoxic EOC cells enhances drug resistance by activating the PTEN/PI3K/AKT pathway and is closely related to the recurrence of EOC (Zhu X. et al., 2019). TAM-derived exosomes also contain miR-365 that upregulates nucleotide triphosphates and induces cytidine deaminase, leading to gemcitabine resistance in PDAC cells (Binenbaum et al., 2018). Furthermore, blocking the transmission of lncRNA SBF2-AS1 via exosomes from M2 macrophage to PC enhances miR-122-5p expression and decreases XIAP expression, which further inhibits PC progression (Yin Z. et al., 2020). Otherwise, exosomes also mediate the close interaction between TAMs and T cells. TAM-released miR-29a-3p and miR-21-5p are incorporated into CD4+ T cells via exosomes, which result in the imbalance of the Treg/Th17 ratio and formation of an immune-suppressive microenvironment, thereby facilitating EOC progression and metastasis (Zhou J. et al., 2018). Thus, these findings suggest that blocking the transfer of these exosomes may provide novel targets for cancer treatment.

## Diagnostic, Prognostic, and Therapeutic Applications of Exosomal ncRNAs in Cancer

With knowledge on the capabilities of circulating exosomes carrying different types of molecules, which can be recognized and absorbed by specific receptors of recipient cells, exosomes derived from stromal cells or tumor cells have been considered as ideal biomarkers and particular carriers for the transmit of functional therapeutic targets to cancer cells. Many studies have demonstrated that ncRNAs transferred through exosomes play vital roles in cancer diagnosis and prognosis, as well as act as predictors and monitors for anti-cancer therapy. Of note, considering the functional roles of ncRNAs in cancer modulation, depleting the delivery of these exosomal nucleic acids or blocking the uptake of exosomes has opened a valid method for cancer therapy. For instance, exosomes transferring anti-cancerous ncRNAs, such as miR-320a (Zhang Z. et al., 2017), miR-148b (Li et al., 2019), and miR-4516 (Kim et al., 2020) to recipient cancer cells, turns out to be a potential treatment strategy for cancer therapy. Interestingly, these miRNAs exhibit absence of expression in CAF-derived exosomes, and the absence promotes cancer progression, indicating that the delivery of these exosomal miRNAs can be used as anticancer drugs. Furthermore, exosomal lncRNA CCAL intrigues oxaliplatin resistance of CRC *in vivo* and *in vitro* (Deng et al., 2020), and exosomal miR-103 increases vascular permeability and tumor metastasis (Fang J. H. et al., 2018). Therefore, these findings suggest that blocking the transportation of exosomal ncRNAs may become potential therapeutic ways for cancer progression and chemoresistance.

The biomarkers in blood or urine can help to identify the presence of cancer and predict survival, metastasis, or treatment response. Their acquisition causes far less invasiveness than that required for tumor biopsy. It has been demonstrated that the expression of circ-PDE8A in the plasma exosomes of PDAC patients is positively associated with lymphatic invasion, TNM stage, and a poor survival rate of PDAC patients and so may become a useful marker for the diagnosis or progression of PDAC (Li Z. et al., 2018). Moreover, the expression of circPRMT5 in serum and urine exosomes of urothelial carcinoma of the bladder (UCB) patients is upregulated and promotes tumor invasion and metastasis, which also can be a prognostic biomarker and a further developable therapeutic target for UCB patients (Chen X. et al., 2018).

A large number of trials have been registered to study the role of exosomes in tumor patient diagnosis, prognosis, and therapy. One recruiting observational study will use RNA sequencing to identify LC-specific exosomal lncRNAs in blood samples from patients with lung cancer (NCT03830619). Another interventional trial aims to find new exosomal small RNA biomarkers for the diagnosis and prognosis of pancreatic cancer using small RNA liquid biopsy, combined with EUS-FNA tissues (NCT04636788). Furthermore, a completed observational trial (NCT03032913) aims at determining whether the presence of onco-exosomes from blood and portal vein blood samples is valid for the diagnosis of PDAC patients with suspicion or recent diagnosis and for disease monitoring on pancreatic ductal adenocarcinoma patients and non-cancer patients. These studies have confirmed that exosomes show excellent potency in clinical applications. Unfortunately, the application of therapeutic exo-ncRNA is still in its infancy, and no relevant trial registration is available currently. It is worth noticing that exosomes secreted by stromal cells in the TME, especially dendritic cell (DC) cells, can be used as vaccines to induce anti-tumor immune responses. There is a phase II observational study aiming to validate an isolation method of second-generation DC-derived exosomes (Dex) and confirm their enhanced immune stimulatory ability (NCT01159288). It proposes a maintenance immunotherapy in advanced unresectable non-small cell lung cancer (NSCLC) patients involving the treatment of metronomic cyclophosphamide (mCTX) followed by vaccinations with tumor antigen-loaded Dex and evaluates the progression-free survival rate (PFS) of these patients in 4 months. Therefore, completion of this trial will hopefully prove exosomal ncRNA biomarkers that can be used in cancer early detection and therapy.

## Conclusion and Perspective

TME is the cellular environment required for malignant tumor progression and metastasis. Emerging research has revealed novel mechanisms of how exosome-based delivery of ncRNAs underlying the intrinsic intercellular communication between tumor and the TME regulates tumor development and progression (Wu et al., 2019b; Kalluri and LeBleu, 2020). As described, tumor-derived exosomal ncRNAs support tumor progression and metastasis by modulating energy metabolism, promoting angiogenesis, and regulating the EMT process, which remodel the surrounding microenvironment (Monaco et al., 2019; Zhou et al., 2019). Stromal cells also release exosomal ncRNAs, which can be transferred to cancer cells, thereby modulating tumor progression (Xu et al., 2019; Yin et al., 2019). Thus, exo-ncRNAs from both tumor and stromal cells have been shown to be useful indicators for tumor initiation and progression, as well as novel approaches for cancer therapy. Due to their capability to be released into biological fluids and transported between diverse cells, exosomes become important carriers for the delivery of drugs and other functional molecules. We have not been able to trace any other trials except the one using DC-exosomes as vaccinations that will evaluate the PFS in NSCLC patients (NCT01159288). However, it does appear that more and more clinical trials targeting exo-ncRNAs could be introduced in the future.

Due to the technological improvement of RNA isolation and sequence analysis, more and more ncRNAs contained in exosomes have been gradually discovered. Exo-ncRNA is easily detected in blood, serum, or urine and other liquids and can provide a non-invasive way to detect the presence of tumors and evaluate the prognosis of patients. However, the physiological and pathological roles of exosomal ncRNAs, especially lncRNAs and circRNAs, in the TME remain to be further explored. In this review, we have found that there are many articles focusing on the relationship between circRNAs and angiogenesis, but few articles relating the detailed function of exosomal cirRNAs secreted from MSCs or MDSCs in tumor angiogenesis. Further work is required to be accomplished, and it is necessary to clarify the potential functional mechanism of exosomes within the TME, so as to provide opportunities for intervention and targeted therapy of exosomal communication systems.

In the future, exo-ncRNAs may become a new type of molecular biomarker to distinguish malignant from benign tumors and to assess the risk of metastasis or chemotherapy drugs. In addition, urine or blood samples of patients for early diagnosis are ideal non-invasive detection samples. More and more studies have confirmed the important role of ncRNA carried by exosomes in tumor cells and animal experiments. The studies on exo-ncRNAs have indeed opened a new chapter, indicating that circulating exo-ncRNAs could possibly act as liquid biopsies and non-invasive biomarkers for the diagnosis and treatment of cancer and other diseases. Although the clinical application is still at the early stage, it is expected that ncRNA-based treatment of cancer patients may be realized in the future.

## Author Contributions

XZ and WX contributed to the conception and design of the study. YqL and YfL performed resource analysis. QC wrote the first draft of the manuscript. All authors contributed to manuscript revision and read and approved the submitted version.

## Conflict of Interest

The authors declare that the research was conducted in the absence of any commercial or financial relationships that could be construed as a potential conflict of interest.

## Abbreviations

TME: tumor microenvironment
MDSCs: myeloid-derived suppressor cells
TAMs: tumor-associated macrophages
EVs: extracellular vesicles
HCC: hepatocellular carcinoma
EMT: epithelial–mesenchymal transition
ECM: extracellular matrix
ECs: endothelial cells.
